# Total Flavones of *Abelmoschus manihot* Remodels Gut Microbiota and Inhibits Microinflammation in Chronic Renal Failure Progression by Targeting Autophagy-Mediated Macrophage Polarization

**DOI:** 10.3389/fphar.2020.566611

**Published:** 2020-09-30

**Authors:** Yue Tu, Qi-Jun Fang, Wei Sun, Bu-Hui Liu, Ying-Lu Liu, Wei Wu, Hong-Yun Yee, Can-Can Yuan, Mei-Zi Wang, Zi-Yue Wan, Ren-Mao Tang, Yi-Gang Wan, Hai-Tao Tang

**Affiliations:** ^1^ Department of Traditional Chinese Medicine Health Preservation, Acupuncture, Moxibustion and Massage College, Health Preservation and Rehabilitation College, Nanjing University of Chinese Medicine, Nanjing, China; ^2^ Department of Traditional Chinese Medicine, Nanjing Drum Tower Hospital, The Affiliated Hospital of Nanjing University Medical School, Nanjing, China; ^3^ Department of Traditional Chinese Medicine, Nanjing Drum Tower Hospital Clinical College of Traditional Chinese and Western Medicine, Nanjing University of Chinese Medicine, Nanjing, China; ^4^ Nephrology Division, Affiliated Hospital of Nanjing University of Chinese Medicine, Nanjing, China; ^5^ Department of Social Work, Meiji Gakuin University, Tokyo, Japan; ^6^ Institute of Huangkui, Suzhong Pharmaceutical Group Co., Ltd., Taizhou, China

**Keywords:** chronic renal failure, total flavones of *Abelmoschus manihot*, gut microbiota, microinflammation, autophagy, macrophage polarization

## Abstract

**Background:**

Recently, progression of chronic renal failure (CRF) has been closely associated with gut microbiota dysbiosis and intestinal metabolite-derived microinflammation. In China, total flavones of *Abelmoschus manihot* (TFA), a component of *Abelmoschus manihot*, has been widely used to delay CRF progression in clinics for the past two decades. However, the overall therapeutic mechanisms remain obscure. In this study, we designed experiments to investigate the renoprotective effects of TFA in CRF progression and its underlying mechanisms involved in gut microbiota and microinflammation, compared with febuxostat (FEB), a potent non-purine selective inhibitor of xanthine oxidase.

**Methods:**

*In vivo*, the CRF rat models were induced by uninephrectomy, potassium oxonate, and proinflammatory diet, and received either TFA suspension, FEB, or vehicle after modeling for 28 days. *In vitro*, the RAW 264.7 cells were exposed to lipopolysaccharide (LPS) with or without TFA or FEB. Changes in parameters related to renal injury, gut microbiota dysbiosis, gut-derived metabolites, and microinflammation were analyzed *in vivo*. Changes in macrophage polarization and autophagy and its related signaling were analyzed both *in vivo* and *in vitro*.

**Results:**

For the modified CRF model rats, the administration of TFA and FEB improved renal injury, including renal dysfunction and renal tubulointerstitial lesions; remodeled gut microbiota dysbiosis, including decreased *Bacteroidales* and *Lactobacillales* and increased *Erysipelotrichales*; regulated gut-derived metabolites, including d-amino acid oxidase, serine racemase, d-serine, and l-serine; inhibited microinflammation, including interleukin 1β (IL1β), tumor necrosis factor-α, and nuclear factor-κB; and modulated macrophage polarization, including markers of M1/M2 macrophages. More importantly, TFA and FEB reversed the expression of beclin1 (BECN1) and phosphorylation of p62 protein and light chain 3 (LC3) conversion in the kidneys by activating the adenosine monophosphate-activated protein kinase-sirtuin 1 (AMPK-SIRT1) signaling. Further, TFA and FEB have similar effects on macrophage polarization and autophagy and its related signaling *in vitro*.

**Conclusion:**

In this study, we demonstrated that TFA, similar to FEB, exerts its renoprotective effects partially by therapeutically remodeling gut microbiota dysbiosis and inhibiting intestinal metabolite-derived microinflammation. This is achieved by adjusting autophagy-mediated macrophage polarization through AMPK-SIRT1 signaling. These findings provide more accurate information on the role of TFA in delaying CRF progression.

## Introduction

Chronic renal failure (CRF) is a global public health problem, as a common sequel in the various types of chronic kidney disease (CKD) (Lv and Zhang, 2019; Smyth et al., 2019). Although there is extensive knowledge regarding CRF progression, the development of successful therapeutic strategies remains a challenge in clinics. Therefore, the discovery of a new treatment to delay CRF progression would be an important step toward achieving improved general kidney health. There are many factors that contribute to CRF progression, including increased production of oxidative stress and acidosis, proinflammatory cytokines, chronic and recurrent infections, altered metabolism of adipose tissue, and last but not least gut microbiota dysbiosis, which is a source of systematic microinflammation *in vivo* (Machowska et al., 2016; Mahmoodpoor et al., 2017; Wang et al., 2020). Currently, investigators concern that gut microbiota dysbiosis and microinflammatory lesions play fundamental roles during CRF progression. The specific uremic milieu in CRF, due to influx of urea and other retained toxins, impairs intestinal barrier function, and induces microinflammation through the intestinal tract; thus, it is vital in shaping gut microbiomes in terms of structure, composition, and function (Anders et al., 2013). Accordingly, gut microbiome diversity is significantly damaged in patients with kidney injury, along with the disorder host homeostasis and confusion of its metabolites, including d-amino acid oxidase (DAO), serine racemase (SRR), d-serine, and l-serine (Nakade et al., 2018). Consequently, targeting gut microbiota dysbiosis and intestinal metabolite-derived microinflammation may be promising adjuvant approaches to protect against kidney injury in CRF.

Macrophages in the kidney are important inflammatory cells and exert multiple biological effects on kidney injury in CKD. Macrophages can be polarized into two distinct functional phenotypes: classically activated macrophages (M1) and alternatively activated macrophages (M2). M1-type macrophages secrete inducible nitric oxide synthase (iNOS) and interleukin 6 (IL6), which induce proinflammatory effects, chemotaxis, and matrix degradation; M2-type macrophages express CD163, CD206, and arginase 1 (ARG1), which play anti-inflammatory, tissue repair, and angiogenesis roles (Lee et al., 2011; Wang and Harris, 2011; Cao et al., 2014). Hence, regulation of macrophage polarization is considered an effective strategy for the anti-inflammatory treatment of CKD.

Autophagy is a highly conserved process, which involves forming autophagosomes that deliver macromolecules and whole organelles or intracellular pathogens to lysosomes for degradation (Mizushima and Komatsu, 2011). Autophagic dysfunction is involved in the pathogenesis of a variety of kidney injuries. Changes in light chain 3 I/II (LC3 I/II), beclin 1 (BECN1), and polyubiquitin-binding protein p62/sequestosome 1 (p62/SQSTM1) are considered markers of autophagy. Autophagy in the kidney is regulated by the major nutrient-sensing pathways, including the mammalian target of rapamycin (mTOR), adenosine monophosphate-activated protein kinase (AMPK), and sirtuin 1 (SIRT1) (Hoyer-Hansen and Jaattela, 2007; Lee et al., 2008; Hosokawa et al., 2009). Moreover, autophagy has been recognized as a crucial effect in macrophage polarization. Chen et al. reported that rapamycin-induced autophagy stimulates inflammation and the differentiation of monocytes into M1 macrophages in humans (Chen et al., 2012). Liu et al. proved that, in high-fat diet and lipopolysaccharide-treated autophagy related 5 (ATG5) knockout mice, autophagy exhibits abnormalities in polarization with both an increase of proinflammatory M1 and a decrease of anti-inflammatory M2 polarization (Liu et al., 2015). Thus, autophagy may modulate the macrophage polarization into M1 or M2 in the kidney.

The total flavones of *Abelmoschus manihot* (TFA) are the main components of Huangkui capsule (HKC; the local name in China), which is a modern Chinese patented medicine preparation extracted from *Abelmoschus manihot* (AM). This preparation has been approved by the China State Food and Drug Administration (Z19990040) for the treatment of CKD and has been used over the past two decades (Zhang et al., 2014; Li et al., 2020). Clinical evidence in China has suggested that HKC (at the safe and effective dose of 7.5 g/kg/day) can reduce urinary albumin in CKD. Our previous studies proved that HKC and its bioactive component hyperoside can alleviate tubular injury in diabetic nephropathy by regulating inflammatory signaling (Han et al., 2019) and in d-galactose-induced aging kidney by adjusting autophagy (Liu et al., 2018). Despite this evidence, important issues remain unresolved in terms of the therapeutic effects of TFA on CRF. For example, whether TFA can alter gut microbiota and microinflammation *in vivo*, and the possible underlying mechanisms involved in this process remain to be determined.

To address these issues, in this study, we used modified CRF rat models induced by uninephrectomy, potassium oxonate, and proinflammatory diet (PD), as well as the RAW 264.7 cells exposed to lipopolysaccharide (LPS) model. We expounded the actions of TFA remodeling gut microbiota dysbiosis and inhibiting intestinal metabolite-derived microinflammation by targeting autophagy-mediated macrophage polarization compared with febuxostat (FEB), a potent non-purine selective inhibitor of xanthine oxidase (Tanaka et al., 2015).

## Materials and Methods

### Extraction and Quality Control of TFA

TFA purchased from Suzhong Pharmaceutical Group Co., Ltd. (Taizhou, China) is composed of extracts from AM. The extraction and production process of TFA, protected by the China Patent Office, are subjected to strict quality control, and the main components are subjected to standardization. In addition, the quality of TFA was examined with a fingerprint analysis shown in the **Supplemental Materials**. And the analysis and detection methods were described in detail in the **Supplemental Materials**. Rutin, hyperoside, isoquercitrin, and quercetin are bioactive components of TFA. In this study, TFA was dissolved in distilled water (TFA suspension) and stored at 4°C prior to use.

### Animals and Treatments

Twenty-six male Sprague–Dawley rats, weighing 200 ± 20 g, were provided by the Animal Center of Nanjing Medical University (Nanjing, China). All animal handling and experimental procedures were performed in accordance with local ethics committees and the National Institutes of Health Guide for the Care and Use of Laboratory Animals. All efforts were made to minimize animal suffering and reduce the number of animals used. All procedures involving animals in this study were approved by the Animal Ethics Committee of Nanjing University Medical School (qualified number: SYXK2014-0052). The feeding conditions of rats were previously described (Han et al., 2019). All rats were divided into four groups, according to a random number table, as follows: five, seven, seven, and seven rats in the Normal, Model, TFA, and FEB groups, respectively. The rats in the Normal group were given distilled water and a standard diet, including 14 kcal% fat, 21 kcal% protein, and 65 kcal% carbohydrates for 11 weeks. The rats in the other three groups were subjected to right nephrectomy, received potassium oxonate at a dosage of 250 mg/kg/day for 7 days by gastric gavage, and given a standard diet in week 1. Subsequently, the rats in the three groups were given PD, D12492 containing 60 kcal% fat, 20 kcal% protein, 20 kcal% carbohydrates, and 0.3% adenine, from 2 to 11 weeks. The experimental process was shown in the **Supplemental Materials**. TFA as the main components of HKC is composed of the extracts from AM. One capsule of HKC contains 0.5 g of AM and 34 mg of TFA. Our previous animal experiment revealed that a 2 g/kg/day dose of HKC, which contains a 136 mg/kg/day dose of TFA, can significantly attenuate renal fibrosis (Wu et al., 2018). Thus, the rats in the TFA group received a TFA suspension at a dosage of 136 mg/kg/day. Meanwhile, the rats in the FEB group were given a FEB suspension at a dosage of 4.11 mg/kg/day, and the rats in the Model group received distilled water by gastric gavage. Potassium oxonate and FEB were obtained from Sigma-Aldrich Chemical Co. (St. Louis, MO, USA).

### Blood and Tissue Sample Collection and Storage

At the end of the experiment, all rats were anesthetized through an intraperitoneal injection of ketamine and diazepam (1:1), and sacrificed by cardiac puncture. The blood samples were centrifuged at 2,500 rpm for 10 min to collect the serum, which was immediately frozen at −80°C for biochemical analysis and enzyme-linked immunosorbent assay (ELISA) assays. The left kidney tissues were dissected for Western blotting (WB) analysis, as well as for immunofluorescence, immunohistochemistry, and histopathological staining.

### Serum Biochemical Parameters and 16S rDNA Amplicon Sequencing Analyses

Blood urea nitrogen (BUN), serum creatinine (Scr), and serum uric acid (SUA) were measured using an automatic biochemical analyzer (Beckman Instruments, Inc., California, USA). In addition, fresh stool samples were collected through stimulation of Sprague–Dawley rats, frozen in liquid nitrogen immediately upon collection, and stored at −80°C until analysis. DNA extraction from stool samples was performed, and the microbiomes were analyzed at Novogene (Beijing, China) on an Ion S5^™^ XL platform (Thermo Fisher Scientific, Waltham, MA, USA). High-quality reads were selected for bioinformatics analysis. Based on a 97% sequence similarity, all valid reads from all samples were clustered into operational taxonomic units. The β-diversity, the variation between the experimental groups, was assessed with non-metric multi-dimensional scaling, unweighted unifrac β-diversity, analysis of similarities, MetaStat analysis, and linear discriminant analysis effect size (LEfSe). The prediction of microbial functions was performed using Tax4Fun. The web-based tool MicrobiomeAnalyst (http://www.microbiomeanalyst.ca) was used for the Tax4Fun analysis (Dhariwal et al., 2017).

### ELISA

The expression levels of DAO, SRR, d-serine, l-serine, interleukin 1β (IL1β), and tumor necrosis factor-alpha (TNF-α) in plasma were measured using ELISA kits according to the instructions provided by the manufacturer (Yifeixue Bio Tech, Nanjing, Jiangsu, China).

### WB Analysis

WB analysis was performed as previously described (Tu et al., 2014; Liu et al., 2018). The levels of IL1β, TNF-α, and nuclear factor-kappa B (NF-κB) p65 were assessed using anti-IL1β (40 kDa, 1:500, abs134098), TNF-α (17 kDa, 1:500, abs131997), and NF-κB p65 (65 kDa, 1:500, abs136523) antibodies, respectively (Absin Bioscience, Shanghai, China). The levels of BECN1 and LC3 I/II were assessed using anti-BECN1 (60 kDa, 1:1,000, #3495) and anti-LC3A/B (16/14 kDa, 1:1,000, #12741) antibodies, respectively (Cell Signaling Technology, Beverly, MA, USA). The levels of the phosphorylated and total proteins for mTOR and SQSTM1/p62 were assessed using anti-phospho mTOR (289 kDa, 1:1,000, #5536), anti-mTOR (289 kDa, 1:1,000, #2983), anti-phospho SQSTM1/p62 (62 kDa, 1:1,000, #13121), and anti-SQSTM1/p62 (62 kDa, 1:1,000, #5114) antibodies, respectively (Cell Signaling Technology). The levels of AMPK were determined using an anti-AMPKα1/2 (62 kDa, 1:500, sc-74461) antibody (Santa Cruz Biotechnology, Santa Cruz, CA, USA). The levels of SIRT1, alpha-smooth muscle actin (α-SMA), and collagen I were assessed using anti-SIRT1 (82 kDa, 1:500, abs135553), anti-α-SMA (42 kDa, 1:500, abs130621), and anti-collagen I (139 kDa, 1:500, abs131984) antibodies, respectively (Absin Bioscience). The levels of iNOS, IL6, and CD163, CD206 ARG1 were assessed using anti-iNOS (130 kDa, 1:500, abs130136), IL6 (24 kDa, 1:500, abs135607), and CD163 (130 kDa, 1:500, abs124249) antibodies, respectively (Absin Bioscience). The levels of CD206 and ARG1 were assessed using anti-CD206 (160 kDa, 1:500, sc-58986) and ARG1 (38 kDa, 1:500, sc-271430) antibodies, respectively (Santa Cruz Biotechnology). The levels of glyceraldehyde-3-phosphate dehydrogenase (GAPDH) were assessed using an anti-GAPDH (36 kDa, 1:5,000, AP0063) antibody (Bioworld Technology Inc., St. Louis Park, MN, USA) as a loading control. The blots were visualized using an enhanced chemiluminescence detection system (Tanon-5200Muilti, Shanghai, China). The ImageJ software (NIH, US, http://rsbweb.nih.gov/ij/index.html) was used to perform the densitometric analysis.

### Immunofluorescence Assay

The tissue samples from the renal cortex for immunofluorescence studies were snap-frozen in precooled n-hexane and stored at −70°C. Frozen sections (thickness: 3 μm) were cut using a cryostat. The frozen sections were blocked with 10% fetal bovine serum in phosphate-buffered saline for 1 h at room temperature. The slides were incubated with primary antibodies iNOS (1:500) and CD163 (1:500) (Servicebio, Wuhan, China) overnight at 4°C, and subsequently incubated for 30 min at 37°C with CY3-conjugated secondary antibodies (1:500) (Servicebio). The sections were counterstained with 4, 6-diamidino-2-phenylindole for nuclear staining. Finally, the immunofluorescence images were analyzed by confocal laser scanning microscopy. Fluorescence intensity was quantitated using Image-Pro Plus 5.0 software (Media Cybernetics, Silver Spring, MD, USA).

### Immunohistochemistry Assay

Immunohistochemistry was performed as previously described (Tu et al., 2017). The renal tissue slides were incubated with primary antibodies against IL6 (1:500), LC3 (1:500), and BECN1 (1:500) (Servicebio), as well as the primary antibodies CD206 (1:500, sc-58986) and ARG1 (1:500, sc-271430) (Santa Cruz Biotechnology) overnight at 4°C. The slices were subsequently incubated with horseradish peroxidase-conjugated anti-rabbit secondary antibodies (1:500) (Servicebio). Changes in the kidneys and the positively stained areas were observed using light microscopy (Olympus, Tokyo, Japan). The percentages of the positive areas were calculated by Image-Pro Plus 5.0 software.

### Histopathological Staining

The tissue samples from the renal cortex were paraffin-embedded and 4-μm sections were used. The sections were routinely dewaxed with xylene, washed with ethanol at all levels, and stained with periodic acid-Schiff and Masson’s trichrome. The fibrosis area in the renal interstitial area was calculated using the Image-Pro Plus software.

### Cell Culture and Treatment

RAW264.7 cells obtained from the Cell Bank of the Chinese Academy of Sciences (Shanghai, China) were cultured in DMEM supplemented with 10% fetal calf serum (FBS; Gibco, Grand Island, NY), 100 U/ml penicillin, and 100 μg/ml streptomycin at 37°C in a humidified 5% CO_2_ atmosphere. For the polarization of M1 and M2 macrophages, the RAW264.7 cells were stimulated with lipopolysaccharide (LPS, 100 ng/ml) with or without TFA (20 μg/ml) and FEB (100 nM) for 24 h. Here, the doses of TFA and FEB were determined by the references of Liu et al. (2017) and Kondo et al. (2019).

### Cell Viability Assessment

Cell viability was detected using CCK-8 assay (Beyotime, Shanghai, China) according to the manufacturer’s instructions. The cells were seeded into 96-well plates at a density of 1 × 10^4^ cells per well, with 100 μl medium. After the cells were incubated for the indicated time, 10 μl of the CCK-8 solution was added to each well, followed by incubation for 2 h at 37°C. The optical density was computed at the absorbance of 450 nm, and the cell viability was calculated. For each of these experiments at least three parallel measurements were carried out.

### Statistical Analysis

The WB assessment was repeated at least thrice independently, and the individual data were subjected to a densitometric analysis. The data are expressed as the means ± standard deviation. The statistical analysis was performed by one-way analysis of variance (ANOVA) on normally distributed data, with LSD *post hoc* test, or non-parametric Kruskal-Wallis if not. A *P* value <0.05 indicated a statistically significant difference.

## Results

### TFA and FEB Improve Renal Injury

**Figure 1:**
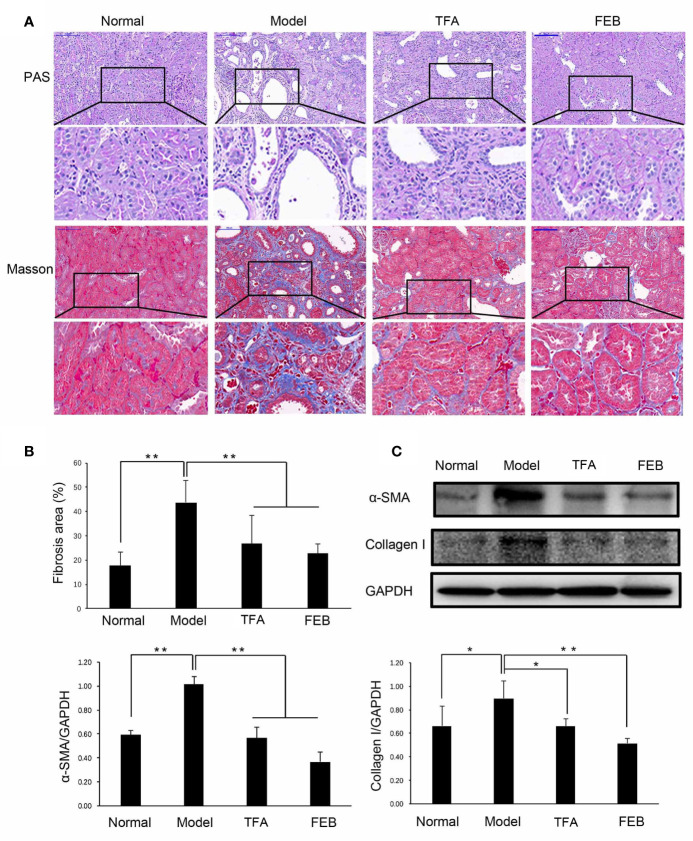
Effects of TFA and FEB on renal injury. **(A)** Photomicrographs of periodic acid-Schiff (PAS) staining and Masson’s trichrome staining in the Normal, Model, TFA, and FEB rat groups. Scale bar: 100 μm. **(B)** Fibrosis area. **(C)** A WB analysis of α-SMA and collagen I in the kidneys of the Normal, Model, TFA, and FEB rats. The data are expressed as the mean ± SD, (n = 3). ^*^ *P* < 0.05, ^**^ *P* < 0.01. TFA, total flavones of *Abelmoschus manihot*; FEB, febuxostat; α-SMA, α-smooth muscle actin; WB, Western blotting; SD, standard deviation.

To assess whether TFA and FEB could ameliorate renal injury, we used the modified CRF model rats induced by right nephrectomy, potassium oxonate, and PD, which is widely used in CRF studies for faithfully recapitulating renal injury-related pathophysiological characteristics, including renal dysfunction and renal fibrosis (Chen et al., 2011; Decleves et al., 2014). First, we examined serum indicators of glomerular filtration function, including BUN, Scr, and SUA in the four rat groups. As shown in **Table 1**, obviously increased levels of BUN, Scr, and SUA in the CRF model rats were detected compared with those noted in the Normal group. Following treatment with TFA or FEB, the levels of BUN, Scr, and SUA in the CRF model rats were significantly decreased compared with those recorded in the Model group. Second, histopathological changes in renal cortices obtained from the four rat groups were observed by light microscopy after staining with periodic acid-Schiff and Masson’s trichrome. Abnormality was found in the Model group, characterized by tubular epithelial cells loss, accumulation of extracellular matrix, and interstitial fibrosis (**Figure 1A**). However, histopathological damages to renal interstitium in the CRF model rats were alleviated by treatment with TFA or FEB (**Figure 1A**). Moreover, the increased percentage of the renal fibrosis area in the CRF model rats was notably reduced in the TFA and FEB groups versus the Model group (**Figure 1B**). Furthermore, evidence from WB showed that the protein expression levels of α-SMA and collagen I in the kidneys of the Model group were significantly increased, and decreased in the TFA and FEB groups, compared with those measured in the Model group, respectively. In short, these results indicated that TFA and FEB can improve renal injury in CRF model rats (**Figure 1C**).

**Table 1:** Blood biochemical parameters in the four rat groups.

Group	BUN (mmol/L)	Scr (μmol/L)	SUA (μmol/L)
Normal	5.56 ± 3.28	15.6 ± 5.03	51.4 ± 22.78
Model	16.17 ± 6.00^**^	82.67 ± 17.77^**^	161.67 ± 23.58^**^
TFA	7.39 ± 3.25^##^	30.71 ± 9.78^##^	39.43 ± 9.66^##^
FEB	4.22 ± 0.52^##^	16.33 ± 5.13^##^	17.67 ± 13.11^##^

The data are expressed as the mean ± SD. ^**^P < 0.01 vs. the Normal group. ^##^P < 0.01 vs. the Model group.

BUN, blood urea nitrogen; Scr, serum creatinine; SUA, serum uric acid; TFA, total flavones of Abelmoschus manihot; FEB, febuxostat; SD, standard deviation.

### TFA and FEB Remodel Gut Microbiota Dysbiosis

**Figure 2:**
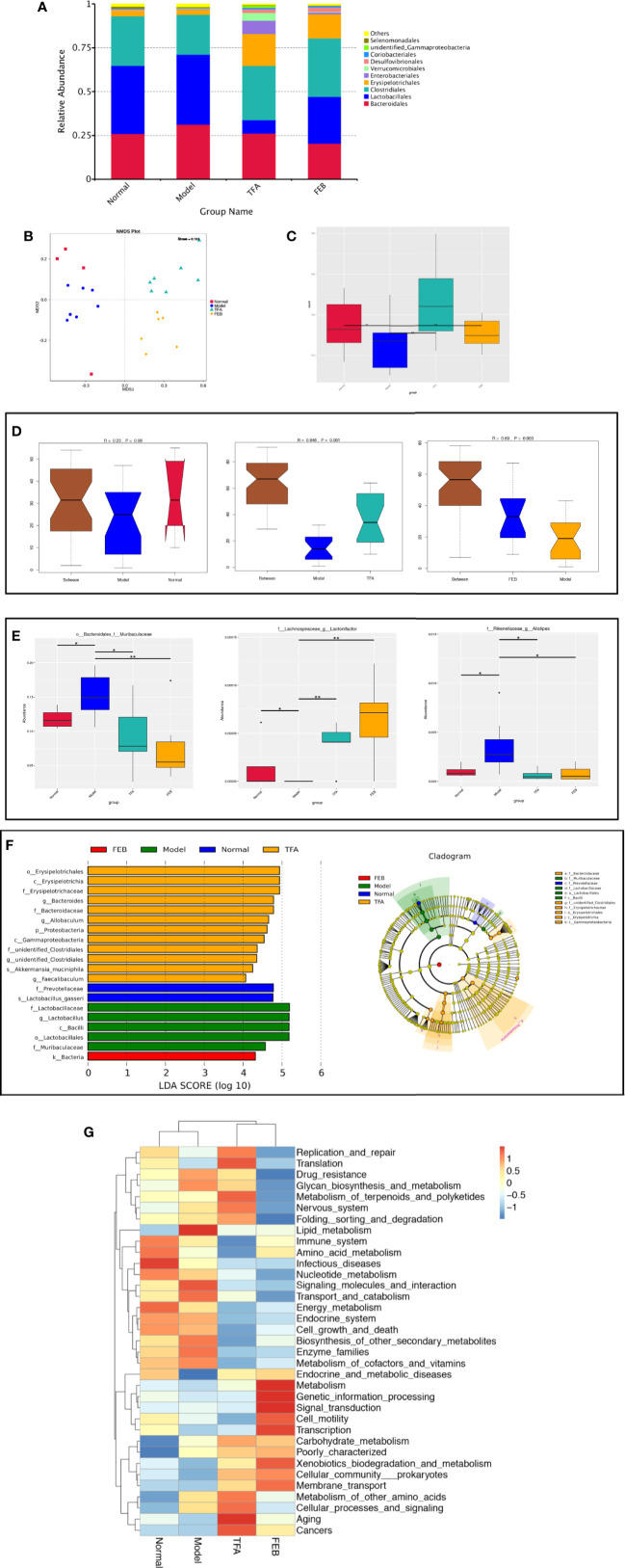
Effects of TFA and FEB on gut microbiota dysbiosis. **(A)** Changes in the relative abundance of gut microbiota at the order level in the four rat groups. **(B)** NMDS analysis for the relative abundance of bacterial OTU in the four rat groups. **(C)** Unweighted unifrac β-diversity of bacterial community. **(D)** ANOSIM analysis of categorical variables between the Normal and Model groups, Model and TFA groups, and Model and FEB groups. **(E)** MetaStat analysis for the relative abundance of significantly altered bacterial taxa at the family and genus levels in the four rat groups. **(F)** LEfSe for the significant bacterial taxa with LDA scores > 4.0 in the four rat groups. Differences are represented by the color of over-represented taxa: blue indicating the Normal group; green indicating the Model group; yellow indicating the TFA group; and red indicating the FEB group. Circles represent phylogenetic levels from phylum (innermost circle) to genera (outermost circle). **(G)** Predicted microbial functions using Tax4Fun in the four rat groups. The data are expressed as the mean ± SD, (n = 3). *P < 0.05, **P < 0.01. TFA, total flavones of *Abelmoschus manihot*; FEB, febuxostat; NMDS, non-metric multi-dimensional scaling; OTU, operational taxonomic unit; ANOSIM, analysis of similarities; LDA, linear discriminant analysis; LEfSe, linear discriminant analysis effect size; SD, standard deviation.

Next, we compared alterations of gut microbiomes in the four rat groups using 16S rDNA amplicon sequencing. Sequences were grouped into provisional clusters as operational taxonomic units. The analysis of changes in the gut microbiome at the order level indicated that the abundance of *Bacteroidales* was increased, while that of *Clostridiales* was decreased in the Model group, compared with those observed in the Normal group (**Figure 2A**). Following treatment with TFA or FEB, the abundances of *Bacteroidales* and *Lactobacillales* were decreased, while that of *Erysipelotrichales* was increased in the TFA and FEB groups, versus the Model group (**Figure 2A**). In **Figure 2B**, Bray–Curtis dissimilarities were calculated and presented by non-metric multidimensional scaling. In terms of unweighted unifrac β-diversity, significant differences in bacterial communities were found between the Normal and Model groups, the Model and TFA groups, as well as the Model and FEB groups (**Figure 2C**). Analysis of similarities for categorical variables revealed that gut microbiomes between the Normal and Model groups were distinct (R = 0.23, *P* = 0.08), and statistical differences between the Model and TFA groups (R = 0.846, *P* = 0.001), as well as the Model and FEB groups (R = 0.69, *P* = 0.003) were significant (**Figure 2D**). Metastat analysis showed that, at the family level, changes of *o_Bacteroidales_f_Muribaculaceae* were obvious between the Normal and Model groups, the Model and TFA groups, as well as the Model and FEB groups. Meanwhile, at the genus level, significant changes of *f_Lachnospiraceae_g_Lactonifactor*, *f_Rikenellaceae_g_Alistipes* were found among these paired groups (**Figure 2E**). **Figure 2F** lists significant changes in microbiota from the phylum level to the species level, as determined by LEfSe. The family *Prevotellaceae* and species *Lactobacillus gasseri* were significantly enriched in the Normal group. The class Bacilli, order Lactobacillales, family *Lactobacillales*, family *Muribaculaceae*, and genus *Lactobacillus* exhibited higher abundances in the Model group. The phylum Proteobacteria, class Erysipelotrichia, class Gammaproteobacteria, order Erysipelotrichales, family *Erysipelotrichaceae*, family *Bacteroidaceae*, family *unidentified Clostridiales*, genus *Bacteroides*, genus *Allobaculum*, genus *unidentified Clostridiales*, genus *Faecalibaculum*, and species *Akkermansia muciniphila* were significantly increased in the TFA group. The kingdom Bacteria was uniquely enriched in the FEB group (**Figure 2F**). In **Figure 2G**, for the Tax4Fun analysis, based on the taxonomical profile, heatmap analysis showed the abundance score of predicted function. These functions were mainly related to infectious diseases, aging, metabolism, genetic information processing, and signal transduction. Taken together, these results indicated that TFA and FEB can remodel gut microbiota dysbiosis *in vivo*.

### TFA and FEB Regulate Gut-Derived Metabolites and Inhibit Microinflammation

**Figure 3:**
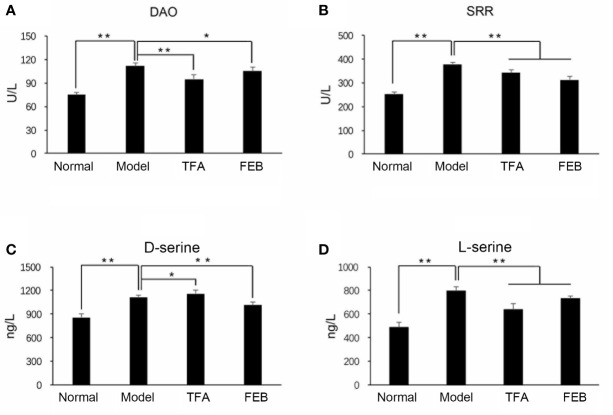
Effects of TFA and FEB on gut-derived metabolites. **(A–D)** Serum levels of DAO, SRR, d-serine, and l-serine in the Normal, Model, TFA, and FEB rat groups. The data are expressed as the mean ± SD. ^*^ *P* < 0.05, ^**^ *P* < 0.01. TFA, total flavones of *Abelmoschus manihot*; FEB, febuxostat; DAO, d-amino acid oxidase; SRR, serine racemase; SD, standard deviation.

A previous study revealed that d-amino acids are produced by gut microbiomes and involved in intestinal mucosal defenses (Sasabe et al., 2016). The levels of d-serine can be regulated by DAO and SRR (Horio et al., 2011; Miyoshi et al., 2011). Therefore, we firstly focused on the metabolisms of DAO and SRR. As shown in **Figures 3A, B**, the serum levels of DAO and SRR in the Model group were significantly increased compared with those noted in the Normal group. After treatment with TFA or FEB, the serum levels of DAO and SRR in the CRF model were significantly decreased compared with those measured in the Model group. Similar to changes observed in the metabolisms of DAO and SRR, the serum levels of d-serine and l-serine in the Model group were also increased, compared with those recorded in the Normal group. However, after treatment with TFA or FEB, we found lower levels of l-serine and d-serine in the TFA and FEB groups, respectively (**Figures 3C, D**).

**Figure 4:**
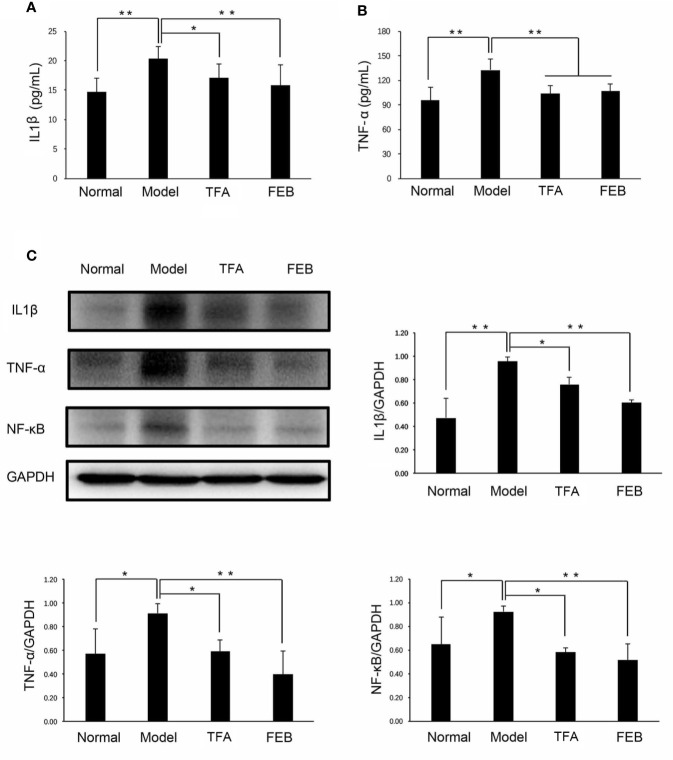
Effects of TFA and FEB on microinflammation. **(A, B)** Serum levels of IL1β and TNF-α in the Normal, Model, TFA, and FEB rat groups. **(C)** A WB analysis of IL1β, TNF-α, and NF-κB in the kidneys of the Normal, Model, TFA, and FEB rats. The data are expressed as the mean ± SD (n = 3), ^*^ *P* < 0.05, ^**^ *P* < 0.01. TFA, total flavones of *Abelmoschus manihot*; FEB, febuxostat; IL1β, interleukin 1β; TNF-α, tumor necrosis factor-α; NF-κB, nuclear factor-κB; WB, Western blotting.

Second, we tested the serum levels of IL1β and TNF-α by ELISA assays to confirm whether TFA and FEB could inhibit microinflammation *in vivo*. **Figures 4A, B** illustrate that the serum levels of IL1β and TNF-α in the Model group were significantly increased compared with those reported in the Normal group. Following treatment with TFA or FEB, the serum levels of IL1β and TNF-α in the CRF model were significantly decreased. Moreover, similar changes in the protein expression levels of IL1β, TNF-α, and NF-κB were found in the kidneys. In comparison with the Model group, the increased protein expression of IL1β, TNF-α, and NF-κB was reversed in the TFA and FEB groups (**Figure 4C**). Collectively, these results indicated that TFA and FEB can regulate gut-derived metabolites and inhibit microinflammation *in vivo*.

### TFA and FEB Modulate Macrophage Polarization *In Vivo* and *In Vitro*

**Figure 5:**
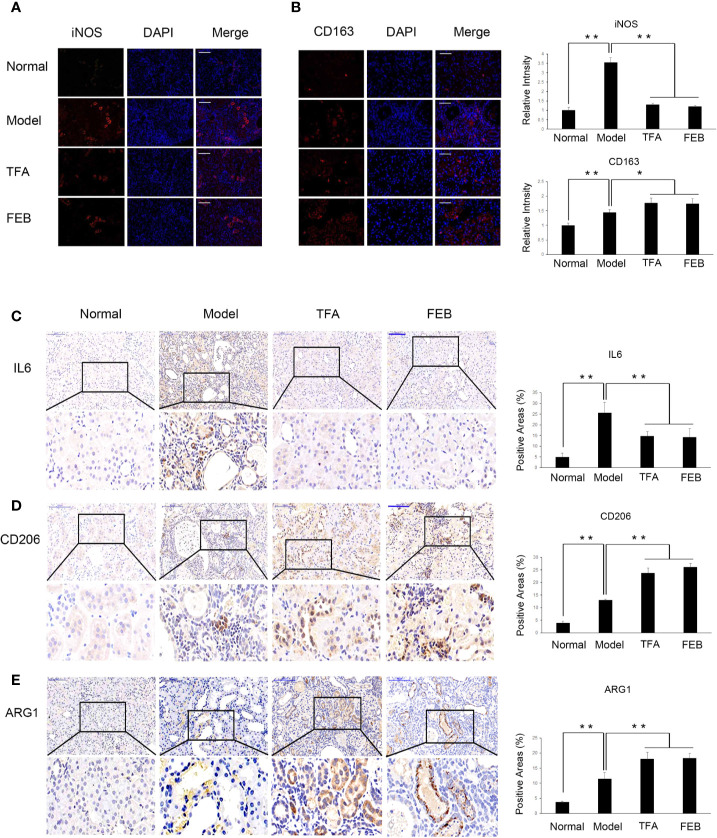
Effects of TFA and FEB on macrophage polarization *in vivo*. **(A, B)** Immunofluorescence staining of iNOS and CD163 in the kidneys of the Normal, Model, TFA, and FEB rats. Scale bar: iNOS, 100 μm; CD163, 50 μm. **(C–E)** Immunohistochemical staining of IL6, CD206, and ARG1 in the kidneys of the Normal, Model, TFA, and FEB rats. Scale bar: 100 μm. *P < 0.05, **P < 0.01. TFA, total flavones of *Abelmoschus manihot*; FEB, febuxostat; iNOS, inducible nitric oxide synthase; IL6, interleukin 6; ARG1, arginase 1.

Inflammatory renal damages and fibrosis are closely related to macrophage polarization in the kidney (Meng et al., 2019). Therefore, we examined the expression of M1 macrophages markers, including iNOS and IL6, as well as M2 macrophages markers (including CD163, CD206, and ARG1) by immunofluorescence and immunohistochemistry staining. As shown in **Figures 5A, C**, our results showed that iNOS and IL6-positive area in the kidneys of the Model group were increased, compared with those observed in the Normal group. After treatment with TFA or FEB, iNOS and IL6-positive area in the kidneys of the CRF model rats were decreased in the TFA and FEB groups, compared with those detected in the Model group (**Figures 5A, C**). Furthermore, in comparison with the Normal group, the expression of CD163, CD206, and ARG1-positive area in the kidneys of the CRF model rats was markedly higher (**Figures 5B, D, E**). After treatment with TFA or FEB, the expression of CD163, CD206, and ARG1-positive area in the kidneys of the CRF model rats was even higher compared with that noted in the Model group’s (**Figures 5B, D, E**).

**Figure 6:**
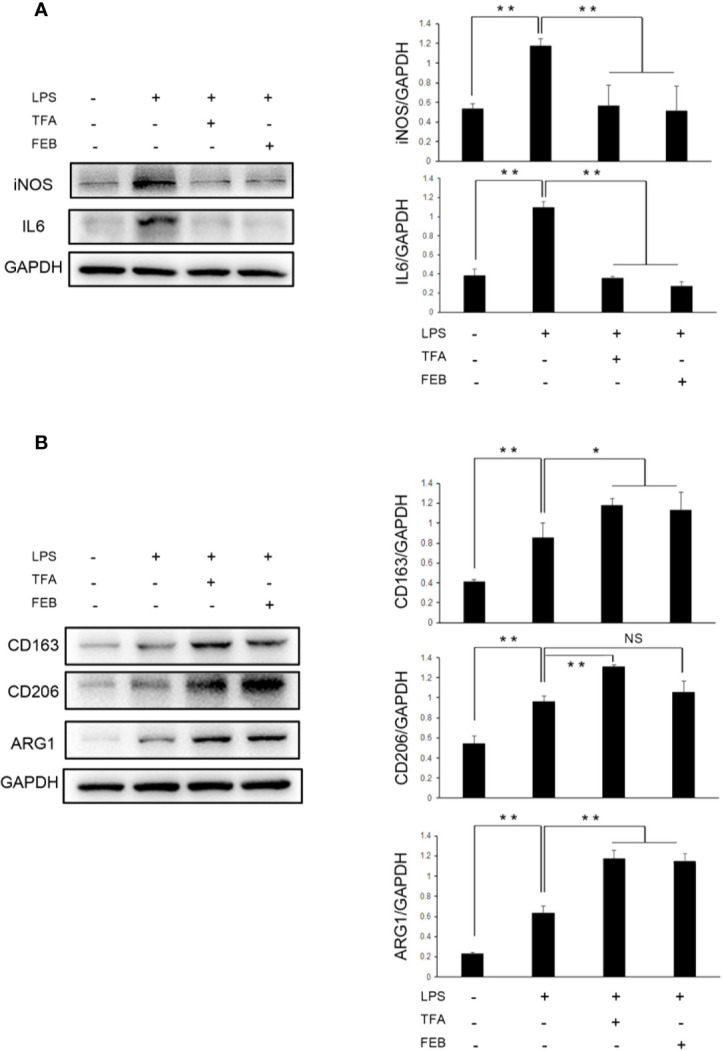
Effects of TFA and FEB on macrophage polarization *in vitro.* **(A)** A WB analysis of iNOS and IL6 in RAW 264.7 cells exposed to LPS (100 ng/ml) with or without TFA (20 μg/ml) or FEB (100 nM) for 24 h. **(B)** A WB analysis of CD163, CD206, and ARG1 in RAW 264.7 cells exposed to LPS (100 ng/ml) with or without TFA (20 μg/ml) or FEB (100 nM) for 24 h. The data are expressed as the mean ± SD, (n = 3). ^*^ *P* < 0.05, ^**^ *P* < 0.01. TFA, total flavones of *Abelmoschus manihot*; FEB, febuxostat; iNOS, inducible nitric oxide synthase; IL6, interleukin 6; LPS, lipopolysaccharide; ARG1, arginase 1; NS, not significant; WB, Western blotting.

Further, to access whether TFA and FEB can induce M1 or M2 polarization *in vitro*, we tested the protein expression levels of M1 and M2 makers in RAW 264.7 cells exposed to LPS with or without TFA or FEB for 24 h. Prior to the formal cellular experiments, the cytotoxicity of TFA or FEB on RAW 264.7 cells was analyzed using CCK-8. As shown in **Supplemental Materials**, the cellular viabilities were significantly decreased under the highest concentrations of TFA at 40 μg/ml and FEB at 200 nM compared to the 20 μg/ml dose of TFA and the 100 nM dose of FEB, respectively. Based on these results, the safe and effective doses of TFA (20 μg/ml) and FEB (100 nM) were selected, respectively. In **Figure 6A**, our results showed that iNOS and IL6 protein expressions were increased in RAW 264.7 cells exposed to LPS, compared to those of the control cells. The protein expression of iNOS and IL6 was decreased in RAW 264.7 cells exposed to LPS with the treatment of TFA or FEB, compared to the treatment with LPS alone. In addition, it is noted that CD163, CD206, and ARG1 protein expressions were increased in RAW 264.7 cells exposed to LPS, compared to those of the control cells. The protein expression of CD163, CD206, and ARG1 was even higher in RAW 264.7 cells exposed to LPS with the treatment of TFA or FEB, compared to the treatment with LPS alone (**Figure 6B**). In sum, these results indicated that TFA and FEB can modulate macrophage polarization both *in vivo* and *in vitro*.

### TFA and FEB Adjust Autophagy-Mediated Macrophage Polarization *In Vivo* and *In Vitro* Through AMPK-SIRT1 Signaling

**Figure 7:**
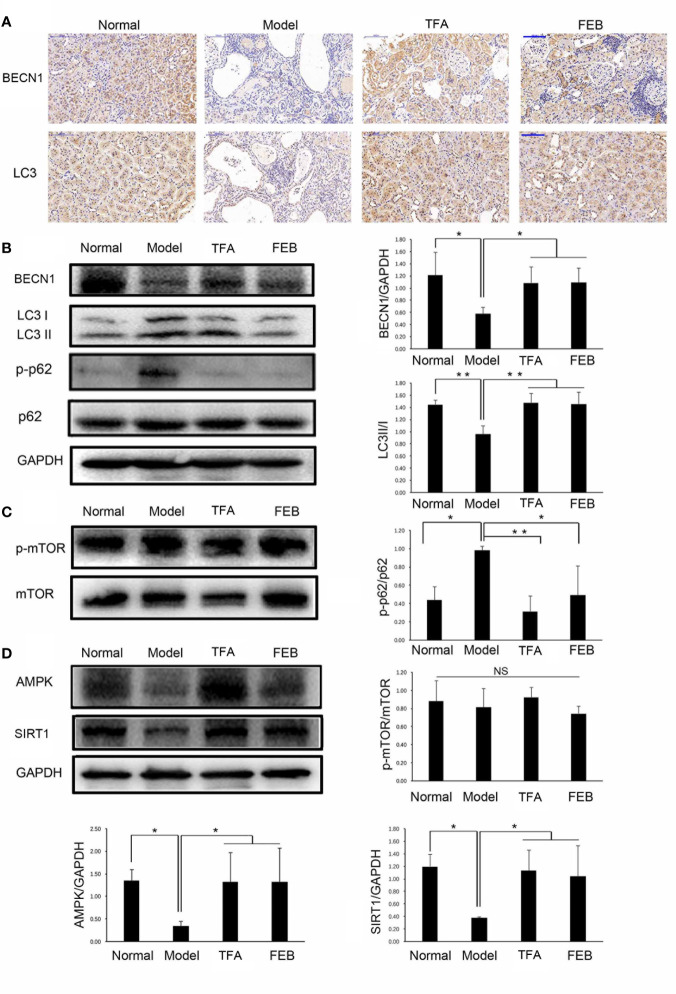
Effects of TFA and FEB on autophagy-mediated macrophage polarization through AMPK-SIRT1 signaling *in vivo*. **(A)** Immunohistochemical staining of BECN1 and LC3 in the kidneys of the Normal, Model, TFA, and FEB rats. Scale bar: 100 μm. **(B–D)** A WB analysis of BECN1, LC3, p-p62, p62, p-mTOR, mTOR, AMPK, and SIRT1 in the kidneys of the Normal, Model, TFA, and FEB rats. The data are expressed as the mean ± SD, (n = 3). ^*^ *P* < 0.05, ^**^ *P* < 0.01. TFA, total flavones of *Abelmoschus manihot*; FEB, febuxostat; BECN1, beclin1; p-p62, phosphorylated p62; p-mTOR, phosphorylated mTOR; NS, not significant; WB, Western blotting.

Autophagy is an important impact factor in macrophage polarization (Chen et al., 2012; Liu et al., 2015). Thus, we evaluated the expression of BECN1 and LC3 in the kidney as the markers of autophagy using immunohistochemical staining. Our results showed that the expression of BECN1 and LC3 in the kidneys of the Model group was lower compared with that measured in the Normal group. Following treatment with TFA or FEB, the expression of BECN1 and LC3 in the kidneys of the CRF model rats were significantly increased (**Figure 7A**). We subsequently tested the protein expression levels of key molecules in autophagy in the kidney by WB analysis. As shown in **Figure 7B**, similar changes in BECN1 protein expression and LC3 conversion (the rate of LC3 II/LC3 I) were confirmed in the four rat groups.

Furthermore, the polyubiquitin-binding protein p62/SQSTM1 is degraded by autophagy (Pankiv et al., 2007). Upregulated protein expression levels of phosphorylated p62 (p-p62) in the kidneys of the Model group were found compared with those recorded in the Normal group. However, the overexpression of p-p62 in the kidneys of the CRF model rats was abolished by treatment with TFA or FEB compared with that observed in the Model group rats (**Figure 7B**). In addition, mTOR signaling is an important regulatory mechanism in autophagy (Munson and Ganley, 2015). Interestingly, there was no significant change in protein expression levels of phosphorylated mTOR and mTOR in the four rat groups (**Figure 7C**). **Figure 7D** shows that the protein expression levels of AMPK and SIRT1 in the kidneys of the Model rats were significantly downregulated compared with those recorded in the Normal rats. In comparison with the Model group rats, TFA and FEB reversed the protein expression levels of AMPK and SIRT1 in the kidneys of the CRF rats.

**Figure 8:**
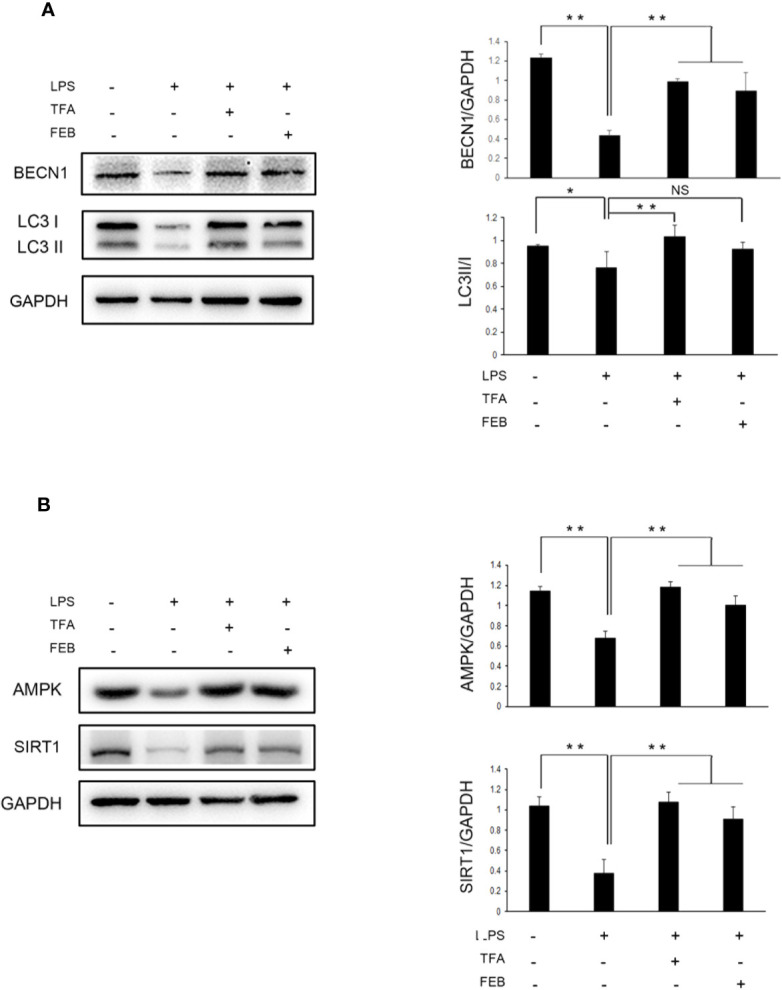
Effects of TFA and FEB on autophagy-mediated macrophage polarization through AMPK-SIRT1 signaling *in vitro*. **(A)** A WB analysis of BECN1 and LC3 in RAW 264.7 cells exposed to LPS (100 ng/ml) with or without TFA (20 μg/ml) or FEB (100 nM) for 24 h. **(B)** A WB analysis of AMPK, and SIRT1 in RAW 264.7 cells exposed to LPS (100 ng/ml) with or without TFA (20 μg/ml) or FEB (100 nM) for 24 h. The data are expressed as the mean ± SD, (n = 3). ^*^ *P* < 0.05, ^**^ *P* < 0.01. TFA, total flavones of *Abelmoschus manihot*; FEB, febuxostat; BECN1, beclin1; LPS, lipopolysaccharide; NS, not significant; WB, Western blotting.

Moreover, the *in vitro* experiments were processed to assess whether TFA and FEB can adjust autophagy-mediated macrophage polarization through AMPK-SIRT1 signaling. Our results showed that the protein expression of BECN1 and LC3 (**Figure 8A**), as well as AMPK and SIRT1 (**Figure 8B**) in RAW 264.7 cells exposed to LPS was significantly downregulated, compared to those of the control cells. In comparison with the stimulation of LPS, the change of LC3 conversion and the protein expression levels of BECN1, AMPK and SIRT1 were upregulated in RAW 264.7 cells exposed to LPS with the treatment of TFA or FEB (**Figures 8A, B**). In brief, these results indicated that TFA and FEB can adjust autophagy-mediated macrophage polarization through AMPK-SIRT1 signaling both *in vivo* and *in vitro*.

## Discussion

To verify the therapeutic effects of natural phytomedicine in CRF progression, it is important to use an appropriate animal model that simulates clinical features similar to those encountered in CRF patients with renal dysfunction and kidney injury. Based on many similar reports (Chen et al., 2011; Decleves et al., 2014), in this study, special multiple methods including right nephrectomy, potassium oxonate administered by gastric gavage, and a PD were jointly used to induce the CRF rat models within 28 days. Potassium oxonate and PD are undeniably the key factors causing hyperuricemia and renal tubulointerstitial damages. Use of uricase inhibitor potassium oxonate is a classic approach to inducing a hyperuricemic animal model, as indicated by drastic increases in SUA levels (Chen et al., 2011). PD is usually utilized as a lipid metabolism-related specific diet for inducing obesity and renal injury (Decleves et al., 2014). In our investigation, significant enlargement of the kidneys with a granular appearance shown in the **Supplemental Materials** was clearly observed in the CRF model rats on day 28. Furthermore, renal tubulointerstitial effects, such as tubular epithelial cells loss, accumulation of extra cellular matrix, and interstitial fibrosis, as well as increased renal α-SMA and collagen I protein expression levels were detected. Furthermore, we noticed that the classic indices of glomerular filtration function, including Scr, BUN, and SUA were significantly elevated. Whereupon, we considered that these modified CRF rat models induced by uninephrectomy, potassium oxonate, and PD should be helpful in unraveling the therapeutic effects and mechanisms of CRF *in vivo*, and discovering new effective drugs against CRF progression.

Gut microbiota dysbiosis has emerged as an important factor conducive to renal dysfunction in CRF progression; alterations of gut microbiomes observed after treatment may be attributable to therapeutic actions (Hand et al., 2016; Wilson and Nicholson, 2017). Yu et al. reported that changes in the gut microbiomes were associated with treatment with allopurinol and benzbromarone in male rats with hyperuricemia, and found that these SUA-lowering drugs caused alterations of gut microbiomes (increased genera *Bifidobacterium* and *Collinsella* and decreased genera *Adlercreutzia* and *Anaerostipes*). Such alterations of gut microbiomes may be indicators of the effectiveness of drug therapy (Yu et al., 2018). In contrast, in our investigation, we found that TFA and FEB ameliorated renal injury and induced changes in the gut microbiomes, including decreased *Bacteroidales* and *Lactobacillales*, and increased *Erysipelotrichales*. Moreover, Metastat and LEfSe analyses showed significant alterations of gut microbiomes at different levels. It should be especially noted that FEB, a novel and selective xanthine oxidase inhibitor, was selected as a positive control drug in this study because FEB exerted dual effects of reducing SUA and regulating the gut microbiome in hyperuricemia (Pascart and Liote, 2019). Thus, we considered that gut microbiota dysbiosis and renal injury in the CRF model rats could be remodeled by treatment with TFA or FEB *in vivo*.

Nakade et al. reported that gut microbiomes may regulate host homeostasis *via* their metabolites (Nakade et al., 2018). In the acute kidney injury animal models, the kidneys were host sites for d-serine metabolism by DAO and SRR on the basis of gut microbiota dysbiosis. In addition, the activity of DAO degrading d-amino acids or d-serine was decreased in the ischemia/reperfusion-injured kidneys. On the contrary, the activity of SRR generating d-serine from l-serine was increased in ischemia/reperfusion-injured kidneys. In our investigation, consistent with the observed renoprotective effect of the gut-derived d-serine *in vivo* (Nakade et al., 2018), we also found that higher levels of d-serine in the CRF model rats were exhibited after treatment with TFA or FEB. Furthermore, the serum levels of DAO, SRR and l-serine in the CRF model rats were significantly increased, and decreased by treatment with TFA or FEB, respectively. On the other hand, recent evidence suggested that the aforementioned gut-derived metabolites released into blood can be linked to systemic microinflammatory responses and play important roles in modulating microinflammation; thus, they are regarded as potential therapeutic targets (Schindler, 2004; Vaziri et al., 2016). In our investigation, TFA and FEB showed anti-inflammatory effects by reducing the secreted levels of IL1β and TNF-α in blood, and downregulating the protein expression levels of IL1β, TNF-α, and NF-κB in the kidneys of the CRF model rats. Consequently, we considered that gut-derived metabolites and microinflammation in the CRF model rats can be regulated and inhibited by treatment with TFA or FEB *in vivo*.

In CRF progression, inflammatory tubulointerstitial lesions are main pathological features in the kidney and involved in macrophages polarization (Wang and Harris, 2011). However, notably, different biological effects of macrophages in renal injury have a relationship with its plasticity and heterogeneity (Mills et al., 2000). Macrophages can be polarized into M1/M2 functional phenotypes. Lu et al. reported that, in obstructed kidneys, significant tubulointerstitial damages and fibrosis are inhibited by quercetin (a natural flavonoid compound) by modulating M1/M2 macrophage polarization. Decreased iNOS and IL12 levels and the proportion of F4/80+/CD11b+/CD86+ macrophages both indicate suppression of M1 macrophage polarization induced by treatment with quercetin in injured kidneys. Interestingly, quercetin also inhibits the polarization of F4/80+/CD11b+/CD206+ M2 macrophages (Lu et al., 2018). In addition, *Sedum sarmentosum* Bunge extract exerts a significant anti-inflammatory effect and alleviates kidney injury by suppressing M1-macrophage polarization marked by downregulating the levels of IL12 and iNOS (Lu et al., 2019). In our investigation, the results revealed that TFA and FEB decreased the levels of iNOS and IL6 (well-defined markers of M1 macrophages), and increased those of CD163, CD206, and ARG1 (generally considered as markers of M2 macrophages in CRF model rats). Therefore, we considered that macrophage polarization in the kidneys of CRF model rats can be modulated by treatment with TFA or FEB *in vivo*. Moreover, macrophage polarization can also be modulated by TFA or FEB in RAW 264.7 cells exposed to LPS *in vitro*.

Autophagy, a protein degradation system, is present at a basal level in all mammals, and regulated by nutrient-sensing-related signaling pathways, such as mTOR, AMPK, and SIRT1 (Lenoir et al., 2016). Liu et al. reported that macrophages in high-fat diet-induced obese mice with defective autophagy, lacking ATG5, have an identical proinflammatory phenotype with elevated M1 and decreased M2 polarization. Thus, autophagy has a critical regulatory function in macrophage polarization that inhibits inflammation (Liu et al., 2015). In our investigation, the results indicated that the lower expression of BECN1 and LC3 in renal interstitium changed the BECN1 and p-p62 protein expression levels and LC3 conversion in the kidneys of CRF model rats. These changes were confirmed and notably reversed by treatment with TFA or FEB *in vivo*, along with alterations of macrophage polarization in the kidney. With the treatment of TFA or FEB, the similar changes of BECN1 protein expression level and LC3 conversion were also observed in RAW 264.7 cells exposed to LPS *in vitro*.

mTOR is the most classical regulatory mechanism in autophagy, which can regulate cell growth, metabolism, survival, and the longevity of organisms (Munson and Ganley, 2015). However, our results showed that mTOR signaling did not exhibit significant changes in the different groups of rats. It is established that, AMPK-SIRT1 is another upstream regulator of autophagy in addition to mTOR. Indeed, in our investigation, we found that the protein expression levels of AMPK and SIRT1 in the kidneys of the CRF model rats, as well as in RAW 264.7 cells exposed to LPS, were markedly downregulated, and reversed by treatment with TFA or FEB. This effect was accompanied by changes in autophagy markers and macrophage polarization. For these reasons, we preliminarily considered that autophagy-mediated macrophage polarization can be adjusted by treatment with TFA or FEB through AMPK-SIRT1 signaling both *in vivo* and *in vitro.*

Finally, the present study had some limitations. First, to explain the complex underlying therapeutic mechanisms of natural phytomedicines on the gut-kidney axis disorder in CRF progression, more classical animal models with renal dysfunction (including the 5/6 nephrectomized model and the unilateral ureteral obstruction-induced model) can be selected in *in vivo* studies. Second, although we have revealed the underlying roles of gut microbiota dysbiosis in this modified CRF rat model, more accurate findings to identify the key bacteria strains that may account for metabolites and microinflammation will be crucial. Third, the metabolism of TFA *in vivo* is complicated; besides gut-derived metabolites, further investigations are required to identify other metabolomics products involved in the relationship between gut microbiota dysbiosis and microinflammatory lesions. Fourth, although TFA affects the activation of the AMPK-SIRT1 signaling pathway *in vivo* and *in vitro*, regrettably we cannot draw a clear conclusion regarding to key signaling molecules of autophagy-related signaling pathways from these two models. A cell sorting *in vitro* and transgenic mouse model using the technique of Cre-Lox recombination *in vivo* may be necessary in future studies to clarify this point. Fifth, based on the classical cognition, LPS can induce M1 polarization, but it cannot affect M2 polarization. However, our results showed that the M2 makers, CD163, CD206 and ARG1 were increased in RAW 264.7 cells exposed to LPS, compared to those of the control cells. Still, there is no denying that, it is not clear what is the reason why M2 polarization is induced in LPS condition. There must be some unidentified factors, and the possible factors will be explored in our future study.

## Conclusion

**Figure 9:**
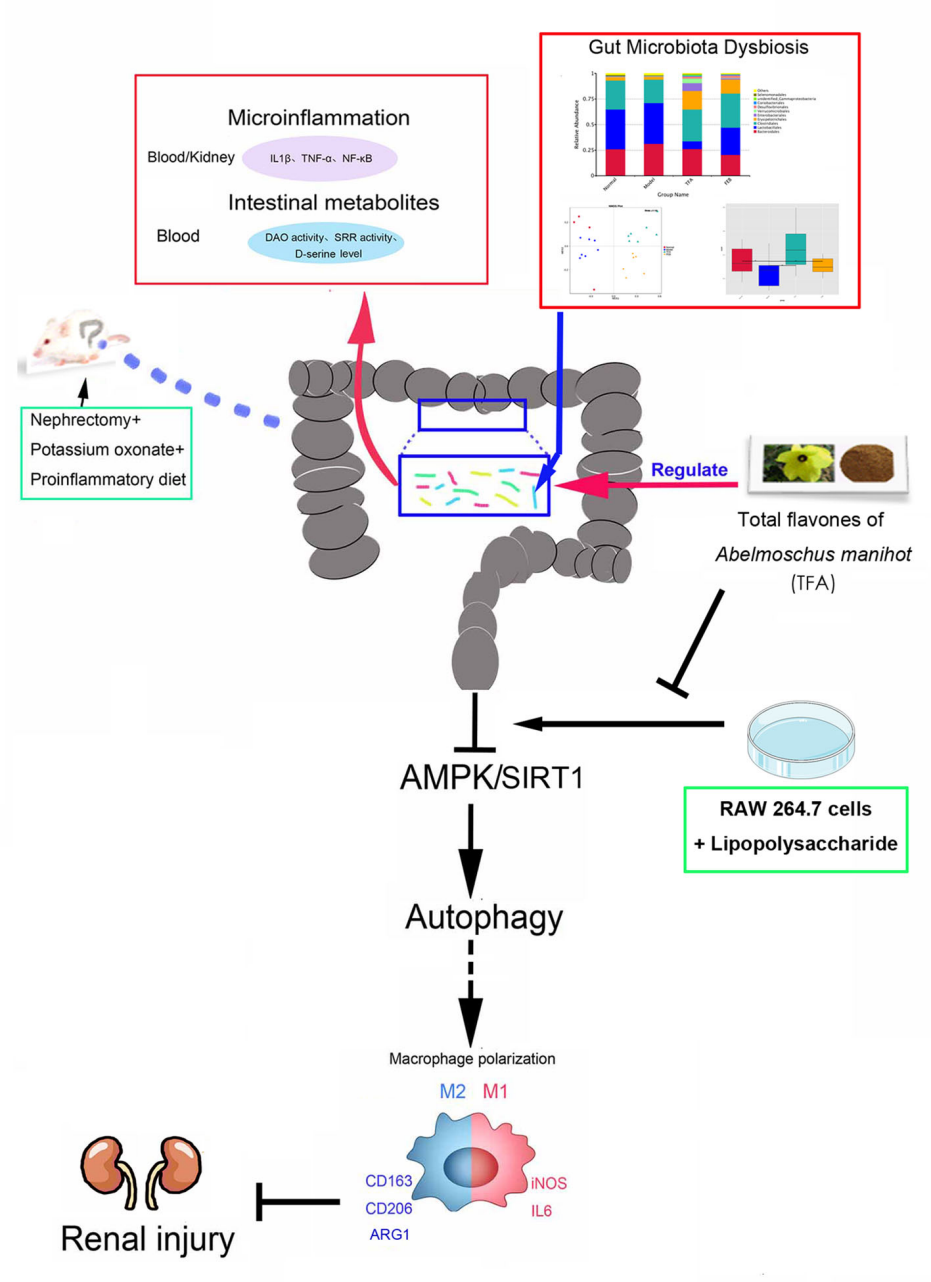
Schematic diagram of TFA remodeling gut microbiota and inhibiting microinflammation in CRF progression. TFA, total flavones of *Abelmoschus manihot*; CRF, chronic renal failure.

In this study, we demonstrated that TFA, similar to FEB, exerts its renoprotective effects partially by therapeutically remodeling gut microbiota dysbiosis and inhibiting intestinal metabolite-derived microinflammation by adjusting autophagy-mediated macrophage polarization through AMPK-SIRT1 signaling (**Figure 9**). These findings provide more accurate *in vivo* and *in vitro* information on the role of TFA in delaying CRF progression.

## Author’s Note

This version is revised by The Charlesworth Group.

## Data Availability Statement

The raw sequences of 24 male rats have been submitted to the NCBI Project under accession number PRJNA600357 with NCBI Sequence Read Archive under accession numbers SRR10866261-SRR10866284.

## Ethics Statement

The animal study was reviewed and approved by: The Animal Ethics Committee of Nanjing University Medical School.

## Author Contributions

Y-GW, WS, and H-TT provided the conception and design of research. YT, Q-JF, B-HL, Y-LL, WW, H-YY, and C-CY performed the experiments. YT, Q-JF, M-ZW, and Z-YW analyzed the data and interpreted the results of experiments. YT prepared the figures. YT and Q-JF drafted the manuscript. Y-GW, H-TT, and R-MT edited and revised the manuscript. YT, Q-JF, and Y-GW approved the final version of manuscript. All authors contributed to the article and approved the submitted version.

## Funding

This study was supported by the National Natural Science Foundation of China (grant numbers: 81603675 and 81573903), Natural Science Foundation of Jiangsu Province for Young Scholars (grant number: BK20161046), Priority Academic Program Development of Jiangsu Higher Education Institutions, Open Projects of the Discipline of Chinese Medicine of Nanjing University of Chinese Medicine Supported by the Subject of Academic Priority Discipline of Jiangsu Higher Education Institutions (grant number: ZYX03KF016), Nanjing Medical Science and Technique Development Foundation (grant number: QRX17042), and Nanjing Famous TCM Doctor’s Studio Programme.

## Conflict of Interest

R-MT and H-TT are employed by Suzhong Pharmaceutical Group Co., Ltd.

The remaining authors declare that the research was conducted in the absence of any commercial or financial relationships that could be construed as a potential conflict of interest.

## Abbreviations

AM, *Abelmoschus manihot*; AMPK, adenosine monophosphate-activated protein kinase; ARG1, arginase-1; ATG5, autophagy related 5; BUN, blood urea nitrogen; CKD, chronic kidney disease; CRF, chronic renal failure; DAO, d-amino acid oxidase; ELISA, enzyme-linked immunosorbent assay; FEB, febuxostat; FBS, fetal calf serum; GAPDH, glyceraldehyde-3-phosphate dehydrogenase; HKC, Huangkui capsule; IL1β, interleukin 1β; IL6, interleukin 6; iNOS, inducible nitric oxide synthase; LC3 I/II, light chain 3 I/II; LEfSe, linear discriminant analysis effect size; LPS, lipopolysaccharide; mTOR, mammalian target of rapamycin; NF-κB, nuclear factor-kappa B; PD, proinflammatory diet; Scr, serum creatinine; SIRT1, sirtuin 1; SRR, serine racemase; SUA, serum uric acid; TFA, total flavones of *Abelmoschus manihot*; TNF-α, tumor necrosis factor-alpha; WB, Western blotting.
