# Supplementary material for: Total Flavones of Abelmoschus manihot Remodels Gut Microbiota and Inhibits Microinflammation in Chronic Renal Failure Progression by Targeting Autophagy-Mediated Macrophage Polarization
Source: Front Pharmacol. 2020 Sep 30;11:566611. doi: 10.3389/fphar.2020.566611 (PMC7554637; doi:10.3389/fphar.2020.566611)

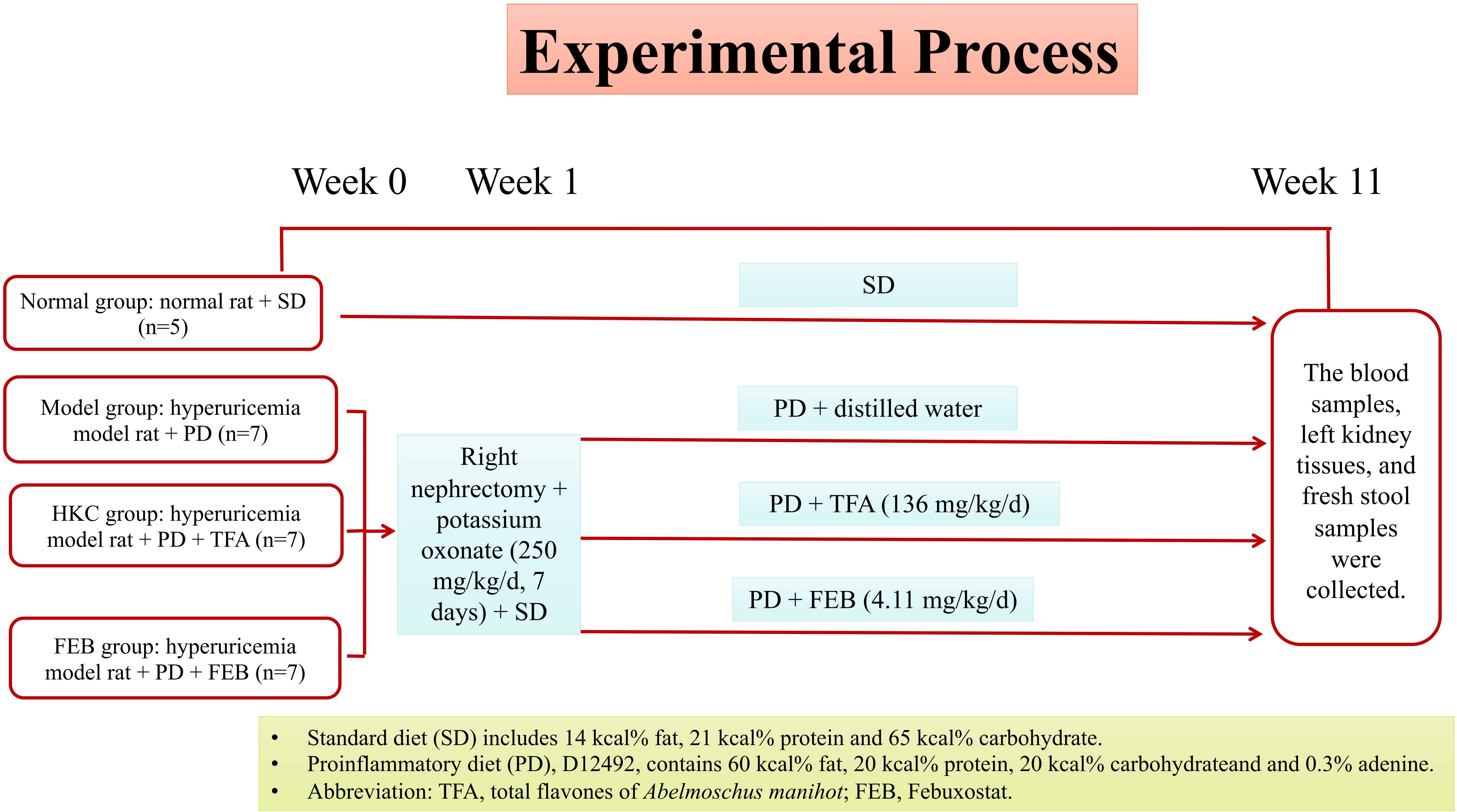
Experimental Process
Week 0
Week 1
Week 11
Normal group: normal rat + SD (n=5)
Model group: hyperuricemia model rat + PD (n=7)
HKC group: hyperuricemia model rat + PD + TFA (n=7)
FEB group: hyperuricemia model rat + PD + FEB (n=7)
Right nephrectomy + potassium oxonate (250 mg/kg/d, 7 days) + SD
SD
PD + distilled water
PD + TFA (136 mg/kg/d)
PD + FEB (4.11 mg/kg/d)
The blood samples, left kidney tissues, and fresh stool samples were collected.
• Standard diet (SD) includes 14 kcal% fat, 21 kcal% protein and 65 kcal% carbohydrate.
• Proinflammatory diet (PD), D12492, contains 60 kcal% fat, 20 kcal% protein, 20 kcal% carbohydrateand and 0.3% adenine.
• Abbreviation: TFA, total flavones of Abelmoschus manihot; FEB, Febuxostat.

TFA Figureprint

Preparation of standard solutions:
Standard stock solutions were prepared by dissolving each standard substance (Rutin, hyperoside, isoquercitrin, and quercetin) in methanol at a concentration of 1 mg/mL. The mixed standard solution of the compounds was prepared by dissolving them in methanol at a concentration of 50 μg/mL. The standard working solutions were prepared by diluting the stock solutions with methanol.

Preparation of samples:
TFA 0.5 g of powdered sample was mixed with 50 mL of methanol in a 100 mL centrifuge tube. After that, the extraction was conducted at room temperature by using ultrasonic extraction for 30 min. The extract was centrifuged at 8000 rpm for 5 min and filtered through 0.22 μm membrane filter. The sample was prepared with the described method for high performance liquid chromatography (HPLC) analysis.

Apparatus and operating conditions:
Chromatographic separation was conducted by using a commercial Agilent ZORBAX SB-C18 analytical column (150 mm×4.6 mm, i.d., 5 μm). The mobile phase contained 0.2% phosphoric acid solution (A) and acetonitrile (B). The initial elution condition was B-A (14:86), linearly changed to B-A (14:86) at 5 min, and then linearly changed to B-A (30:70) at 30 min (Table1). The flow rate was 1.0 mL/min, column temperature kept at 30 °C, the injection volume was 5 μL and the detection wavelength was set as 360 nm.

TFA Figureprint

Table 1. The gradient elution for chromatography analysis

Time (min)	Acetonitrile (B，%)	0.2% phosphoric acid (A, %)
0	14	86
5	14	86
30	30	70

TFA Figureprint

Figure 1. Chromatograms of TFA

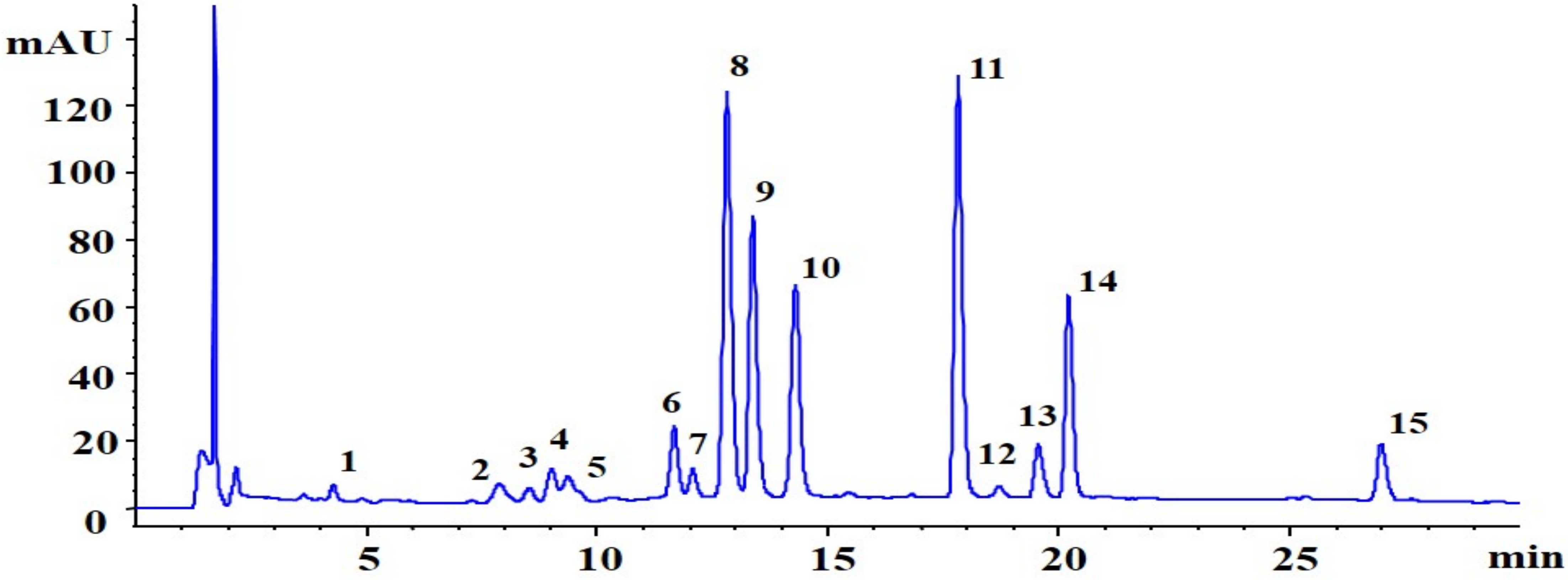

TFA Figureprint

Figure 2. The ultraviolet spectrum of the fifteen compounds in TFA

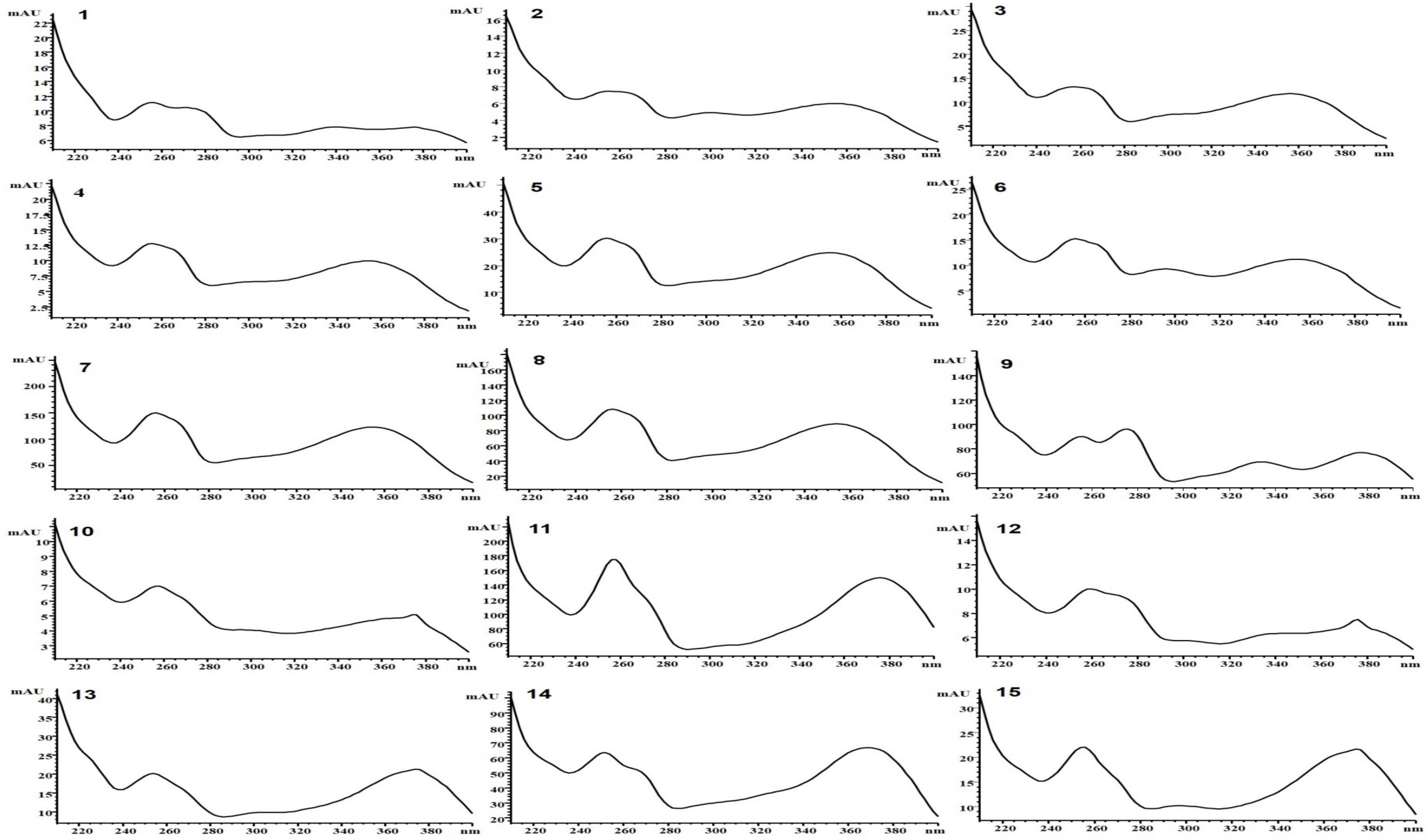

TFA Figureprint

Figure 3. Chromatograms of the mixed standards (a) and the samples of TFA (b)

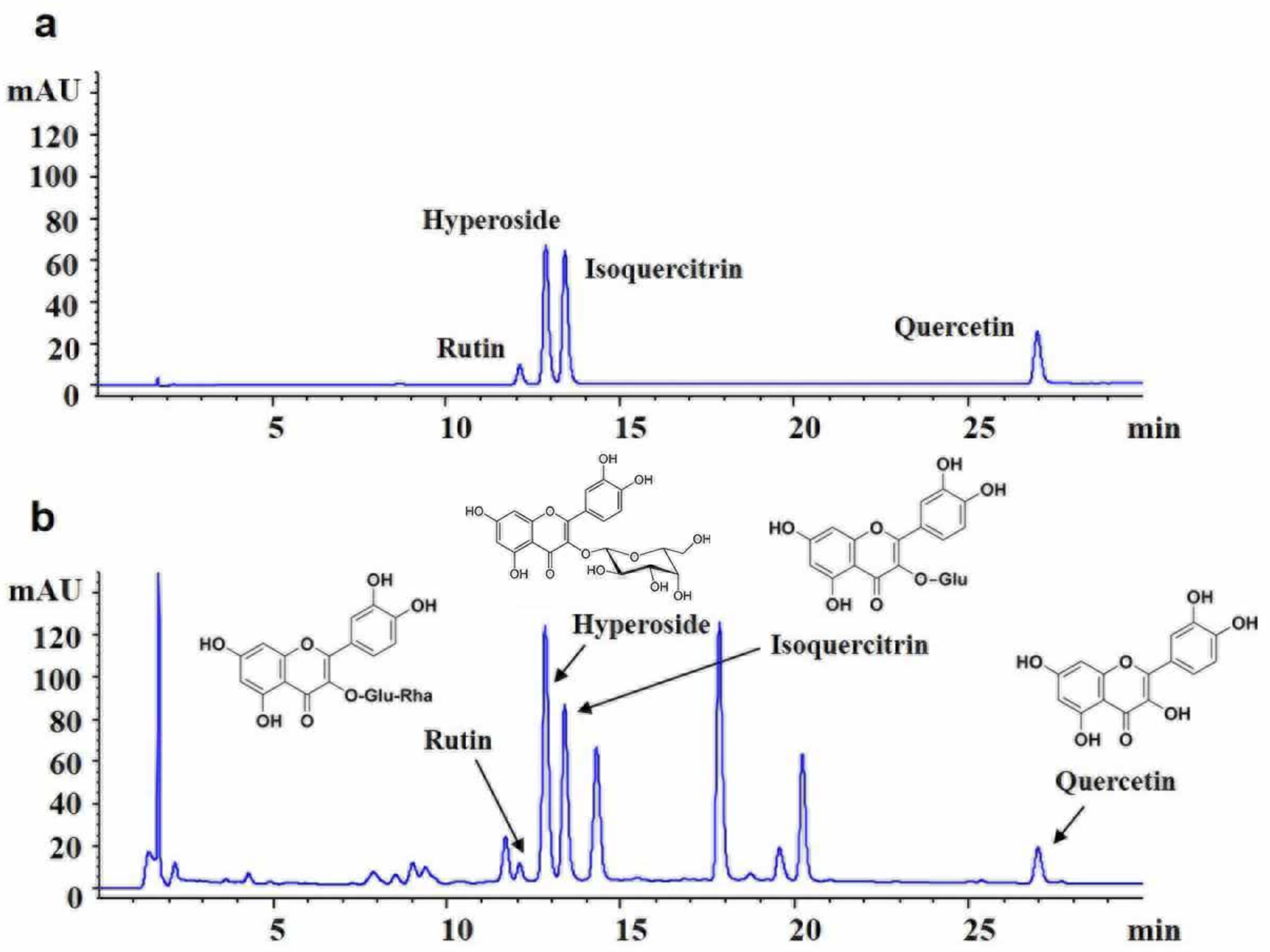

The Appearance of Kidneys in 4 Groups

Normal

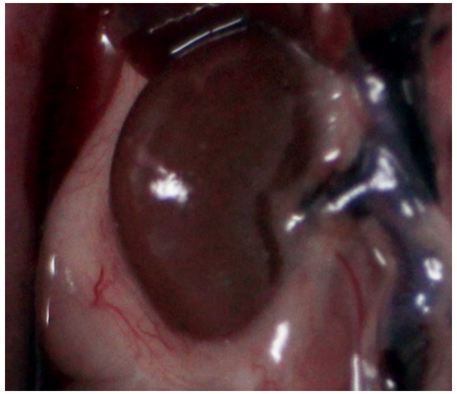

Model

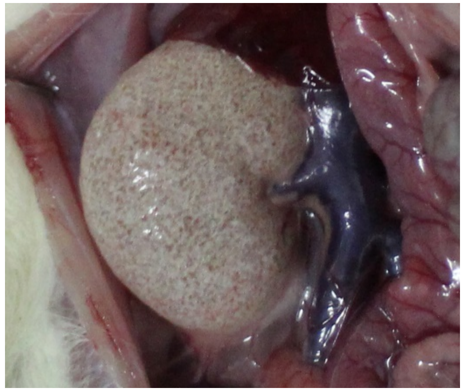

TFA

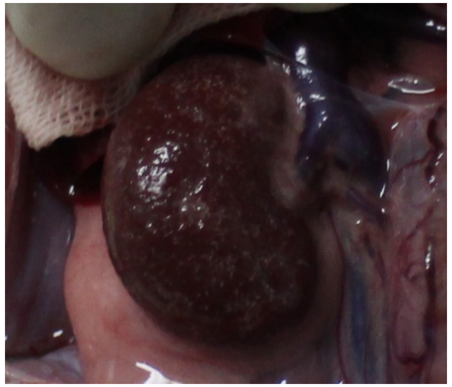

FEB

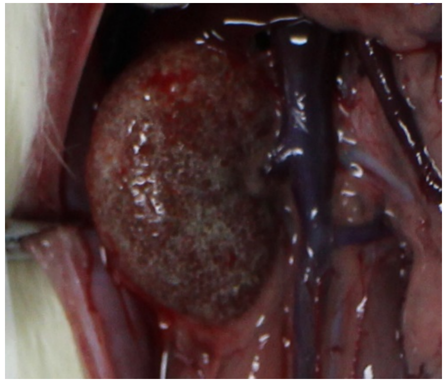

Cell Viability

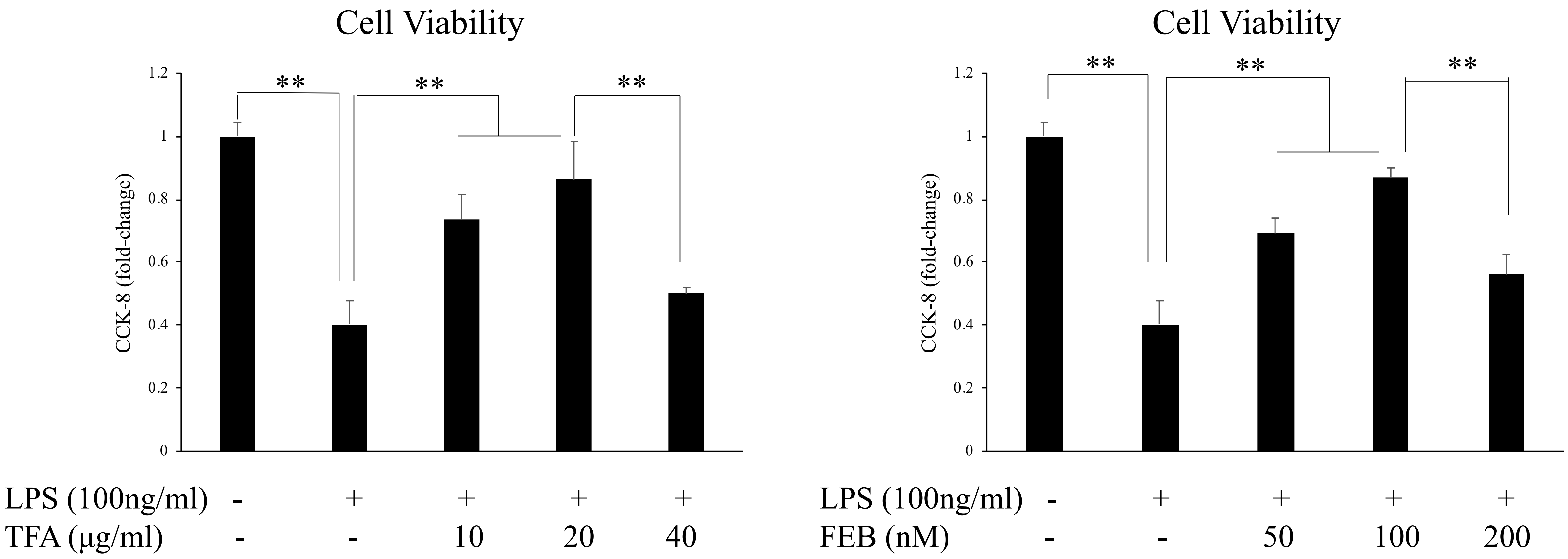

Supplement: Supplementary file 1 [file DataSheet_1.pdf]
